# Effects of Coping-Related Traits and Psychophysiological Stress Responses on Police Recruits’ Shooting Behavior in Reality-Based Scenarios

**DOI:** 10.3389/fpsyg.2019.01523

**Published:** 2019-07-03

**Authors:** Laura Giessing, Marie Ottilie Frenkel, Christoph Zinner, Jan Rummel, Arne Nieuwenhuys, Christian Kasperk, Maik Brune, Florian Azad Engel, Henning Plessner

**Affiliations:** ^1^Institute of Sports and Sports Sciences, Heidelberg University, Heidelberg, Germany; ^2^Department of Sport, University of Applied Sciences for Police and Administration of Hesse, Wiesbaden, Germany; ^3^Psychological Institute, Heidelberg University, Heidelberg, Germany; ^4^Department of Exercise Sciences, University of Auckland, Auckland, New Zealand; ^5^Department of Internal Medicine I and Clinical Chemistry, Steroid Laboratory, Heidelberg University Hospital, Heidelberg, Germany; ^6^Department of Internal Medicine I and Clinical Chemistry, Central Laboratory, Heidelberg University Hospital, Heidelberg, Germany

**Keywords:** performance under stress, police officers, anxiety, cortisol, alpha-amylase, cardiac vagal activity

## Abstract

Police officers are often required to perform under high-stress circumstances, in which optimal task performance is crucial for their and the bystanders’ physical integrity. However, stress responses, particularly anxiety and increased cortisol levels, shift attention from goal-directed to stimulus-driven control, leaving police officers with poor shooting performance under stress. Cardiac vagal activity and coping-related traits (i.e., self-control, sensation seeking) might help individuals to maintain performance under stress. So far, only few studies have integrated coping-related traits, psychophysiological stress markers and occupationally meaningful measures of behavior to investigate police officers’ work performance under stress. Therefore, the present study investigated 19 police recruits (*M_*age*_* = 22.84, *SD* = 3.30) undergoing a reality-based shooting scenario in two experimental conditions in a within-design: low stress (LS) against a non-threatening mannequin, and high stress (HS), involving physical threat by an opponent. Psychological (i.e., anxiety, mental effort) and physiological stress responses (i.e., salivary cortisol, alpha-amylase, cardiac vagal activity) as well as shooting accuracy were repeatedly assessed. It was hypothesized that under stress, police recruits would demonstrate elevated psychophysiological stress responses and impaired shooting performance. Elevated psychophysiological stress responses would negatively influence shooting performance, whereas self-control, sensation seeking and cardiac vagal activity would positively influence shooting performance. While recruits reported significantly higher anxiety and mental effort in the HS scenario, both scenarios elicited comparable physiological responses. Overall, shooting accuracy was low and did not significantly decrease in the HS scenario. Shooting performance was predicted by self-control in the LS scenario and by post-task cardiac vagal activity in the HS scenario. While increased anxiety hints at a successful stress manipulation, physiological responses suggest similar stress levels for both scenarios, diminishing potential behavioral differences between the scenarios. Performance efficiency decreased under stress, as indicated by increasing mental effort. Findings on self-control suggest that suppressing negative stress responses might lead to impaired goal-directed attention, resulting in performance decrements. For police research and training, high-realism scenarios afford an opportunity to investigate and experience psychophysiological stress responses.

## Introduction

On duty, police officers often encounter threatening situations which are accompanied by high levels of acute stress (Anderson et al., 2002). Activities like arresting suspects, responding to general and domestic disturbances or – in more severe cases – using fire weapons impose high stress on the officers and can sometimes be life-threatening. In case of performance failures, police shootings can have tremendous consequences for the officers themselves, colleagues, suspects or innocent bystanders. However, good shooting techniques alone do not appear sufficient for performing well under stressful circumstances: In training situations, hit rates reach 90%, whereas in real-life shootings they do not exceed 50% (Morrison and Vila, 1998; Timmer and Pronk, 2011). Therefore, it is essential to understand how stress and its psychophysiology impacts performance.

Stress results from the individual’s perception of a discrepancy between the demands being placed by the environment and coping resources present in a particular situation (Lazarus and Folkman, 1984). Police officers are often required to respond to situations which threaten their physical integrity or psychological well-being, e.g., spotting a stolen vehicle, a high speed chase or conflict with a suspect (Anderson et al., 2002). These critical incidents are sudden, powerful events that are likely to overwhelm the police officers’ coping resources and to be perceived as outside of their immediate control. When an individual perceives the environmental demands as succeeding its coping resources, a negative, unpleasant psychological state of stress ensues, commonly accompanied by anxiety (Lazarus and Folkman, 1984). Critical incidents in police work hold high levels of novelty, uncontrollability and personal as well as others’ threat of injury or death. Given these characteristics, the body responds to these external demands by an activation of the fast reacting sympathetic adrenomedullary system (SAM; Nater and Rohleder, 2009) and the slower hypothalamo-pituitary-adrenal (HPA) axis (McEwen, 1998; Dickerson and Kemeny, 2004). Although the short-term activation of the systems might be adaptive to maintain bodily homeostasis (the so-called allostasis; Sterling and Eyer, 1988), chronically increased or dysregulated allostasis (referred to as allostatic overload) can lead to disease (McEwen, 1998). Given the regular encounter with critical incidents and the high level of organizational stress, police officers might be at risk for allostatic overload (Anderson et al., 2002, 2016a). Indeed, several studies have shown that critical incidents place high physiological demands on police officers, as measured by salivary cortisol (sCorti) and salivary alpha-amylase (sAA) as surrogate markers of the activation of the HPA and SAM system (Nater and Rohleder, 2009). Although physiological response patterns were inconsistent across studies, it was found that police officers showed increases in subjective stress, heart rate, sCorti and sAA in response to various simulated police scenarios (Regehr et al., 2008; Taverniers and De Boeck, 2014; Strahler and Ziegert, 2015). Groer et al. (2010) compared physiological responses during two virtual reality scenarios of different intensity: the lengthy chase of an armed suspect produced the largest responses in sCorti and sAA, while sAA, but not sCorti was increased during the short chase of a motorcyclist (Groer et al., 2010).

Besides the activation of the HPA and SAM system, heart rate variability (HRV) provides further insight into how people react to stress and perform under stress (Mosley et al., 2018). HRV, defined as the time interval between successive heart beats, represents the cardiac vagal activity, that is the contribution of the parasympathetic nervous system to cardiac function (Laborde et al., 2017). The neurovisceral integration model (Thayer et al., 2009) assumes that cardiac vagal activity indexes an individual’s ability to self-regulate through the organization of physiological resources within central-peripheral neural feedback mechanisms. Higher cardiac vagal activity allows higher adaptability and greater behavioral flexibility in demanding environments (Thayer et al., 2009). Indeed, police officers showed increases in cardiac vagal activity (indicated by high-frequency HRV) in simulated high stress scenarios when facing physical threats compared to medium stress scenarios (Brisinda et al., 2015). Linking cardiac vagal activity to performance under stress, Thompson et al. (2015) found that a smaller reduction in cardiac vagal reactivity (from baseline to task) was associated with better performance in a shooting task.

Stress responses, particularly anxiety and elevated cortisol levels, have been associated with impairments in goal-directed behavior (Eysenck et al., 2007; Schwabe and Wolf, 2011). According to the attentional control theory (Eysenck et al., 2007), anxiety impairs attentional control by increasing the influence of the stimulus-driven system. This impairment leads to an attentional bias to external and internal goal-irrelevant, threat-related information and reduces the level of attention devoted to the current task goals (Eysenck et al., 2007). Initially developed to explain the effect of anxiety on cognitive tasks, the attentional control theory also applies to perceptual motor tasks (Nieuwenhuys and Oudejans, 2012, 2017). Besides anxiety, increased cortisol levels were also shown to influence cognitive functions through a shift from goal-directed control to stimulus-driven behavior under acute stress (Hermans et al., 2014), resulting in performance decrements in perceptual motor tasks (Doan et al., 2007; Cook and Crewther, 2012; Lautenbach et al., 2014). In contrast, Regehr et al. (2008) found that greater cortisol release in response to a reality-based police scenario was associated with higher levels of performance in police recruits. Importantly, stress responses might only impair the efficiency, not necessarily the effectiveness of performance. The attentional control theory postulates that performance decrements might potentially be overcome by the execution of additional mental effort (Eysenck et al., 2007; Nieuwenhuys and Oudejans, 2017).

Based on real-life data demonstrating performance decrements in shooting accuracy under stress (Morrison and Vila, 1998; Timmer and Pronk, 2011), research has evolved to investigate the mechanisms underlying these decrements. Officers reported to be more anxious under stressful circumstances and performed worse in a handgun shooting task, although they reported to invest extra mental effort (Oudejans, 2008; Nieuwenhuys and Oudejans, 2010, 2011). In line with the ACT, gaze behavior of these officers in high stress circumstances revealed that they focused more on threat-related, task-irrelevant stimuli (e.g., opponent’s gun or face) than they did in the low stress condition (Nieuwenhuys and Oudejans, 2010, 2011). Similarly, Vickers and Lewinski (2012) showed that elite police officers had more fixations on task-relevant locations (e.g., locations where a gun could be hidden) and prepared earlier for shooting than novices, resulting in better shooting performance. In contrast, novices even shifted their gaze away from the opponent to their own gun during shooting (Vickers and Lewinski, 2012). Given the importance of continuous visual information input during aiming and shooting, this finding indicates that efficiency of attentional control decreased and thereby, impaired perceptual-motor performance (Nieuwenhuys and Oudejans, 2010).

Although it seems well documented that stress generally impairs performance, people may be distinguished based on their ability to maintain or even increase performance under stress (Geukes et al., 2012). Possibly, variation in performance under stress can be explained by individual differences in task-relevant personality traits that become activated depending on the presence of trait-relevant situational cues (Tett and Gutermann, 2000). In case of extreme stress situations, literature has shown that self-control (Englert and Bertrams, 2015; Landman et al., 2016) and sensation seeking (Zuckerman, 1994; Frenkel et al., 2018; Frenkel et al., unpublished) affect psychophysiological stress responses and performance.

Considering the high risk to the physical integrity in police work, sensation seeking, defined as “seeking of varied, novel, complex, and intense sensations and experiences, and the willingness to take physical, social, legal and financial risks for the sake of such experience” (Zuckerman, 1994, p. 27) might be a relevant personality trait for police performance under stress. According to the psychobiological model (Zuckerman, 1994, 1996), individuals differ in their reaction to novel and intense stimulation: Low sensation seekers (LSS) are overwhelmed by intense stimulations, resulting in attenuated responses and the experience of aversive reactions, such as anxiety (Frenkel et al., 2018). In contrast, high sensation seekers (HSS) experience intense stimulations as pleasant and rewarding, which increases central nervous activity, allowing them to cope efficiently. HSS used more stress-reducing coping strategies (Tschiesner, 2012) and showed attenuated cortisol responses to stress (Couture et al., 2008; Frenkel et al., 2018).

Another trait associated with performance under stress is self-control, generally defined as the ability to willingly exert control over the self by the self (Muraven and Baumeister, 2000). Self-control has been shown to have facilitative effects on perceptual-motor performance under stress as it can help to buffer the negative effects of anxiety. Incorporating the ideas of the ACT (Eysenck et al., 2007), Englert and Bertrams (2015) concluded that self-control enables individuals to counteract the attention disruption under stress in order to obtain performance (Englert et al., 2015a,b). Supporting these theoretical considerations, police officers with high self-control did experience anxiety and increased HR in stressful circumstances, but were able to maintain shooting accuracy (Landman et al., 2016). Instead of utilizing extra effort to suppress or down-regulate their emotional responses, officers with high self-control appear to invest extra mental effort to maintain a goal-directed focus.

Since it is virtually impossible to predict and unethical to manipulate stressful encounters with hand guns in real life, knowledge on police officers’ psychophysiological stress responses and shooting performance in critical incidents is limited (Regehr et al., 2008; Taverniers and De Boeck, 2014; Strahler and Ziegert, 2015). One approach toward the study of psychophysiological stress responses in such situations is to examine police officers’ shooting performance during simulated high-realism shooting scenarios. Following Baumeister et al. (2007) call for more research involving directly observable behaviors in real-world psychological research, the present study applied a reality-based shooting scenario in two conditions: low stress (LS) against a non-threatening mannequin, and high stress (HS), involving physical threat by an opponent who occasionally shot back using colored soap cartridges. Police recruits reported their perceived anxiety as well as the mental effort after each scenario. sCorti and sAA were examined as physiological markers of HPA axis and SAM reactivity, respectively. HRV as index of cardiac vagal activity was continuously monitored throughout the study. Recruits’ behavioral responses were assessed by shooting accuracy. To the best of our knowledge, so far, no empirical study has integrated measures of intra-individual changes in emotional and cognitive stress responses, various biopsychological stress markers and shooting performance. Therefore, the study had two aims: (1) to examine the psychological, physiological, and behavioral stress responses of police recruits in reality-based scenarios and (2) to examine the effects of psychophysiological stress responses and coping-related personality traits on shooting performance. It was hypothesized that:

*Hypothesis 1*: Police recruits would experience elevated stress responses in the HS scenario as compared to the LS scenario (i.e., increases in anxiety, mental effort, sCorti as well as sAA and reduced cardiac vagal reactivity).
*Hypothesis 2*: Shooting accuracy would decrease in the HS scenario as compared to the LS scenario.
*Hypothesis 3*: Resting cardiac vagal activity and smaller reduction of cardiac vagal activity from baseline to task would be positively associated with shooting accuracy.
*Hypothesis 4:* Higher levels of sensation seeking would be associated with reduced stress responses and higher shooting accuracy.
*Hypothesis 5*: Higher levels of dispositional self-control would be associated with reduced stress responses and higher shooting accuracy.

## Materials and Methods

The study design was approved by the ethics committee of the Faculty of Behavioural and Cultural Studies, Heidelberg University, Germany. Written informed consent was obtained from the participating police recruits after receiving detailed information about the design of the study, including the potential risks and benefits. Participants received no financial compensation. Given the involvement of firearms, the present study was executed under the responsibility of certified police firearms instructors, following their standard safety protocol.

### Participants

Data reported here stems from a sample of third-semester police recruits. Overall, 19 German students (3 women, 16 men) participated during two training sessions. Participants were between 19 and 33 years old (*M*_*age*_ = 22.84, *SD* = 3.30). All recruits have already been on duty for 2 months in the riot police and were licensed to carry their handgun on duty. None of them has fired at a suspect or has been shot at.

Participants’ BMI ranged from 21.22 to 28.40 kg/m^2^ (*M* = 24.63, *SD* = 2.02). Eight participants reported to be smokers, but did not differ in physiological measures (i.e., sCorti and sAA). Participants did not report any current or chronic medical or psychiatric diseases. However, one participant was on medication containing cortisone and was therefore excluded from sCorti analyses. One woman took hormonal contraceptives, but her sCorti levels were normal and therefore included in the analyses. The women not taking contraceptives (*n* = 2) were tested during the luteal phase, as indicated by self-report.

### Tasks

Each police officer participated in a 1-h session that involved undergoing the LS scenario first and then the HS scenario.

**FIGURE 1:**
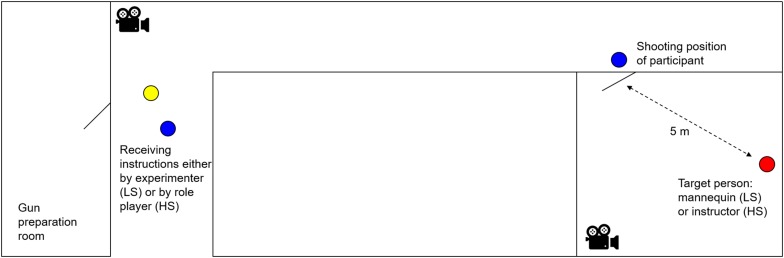
Experimental set-up of the scenarios. Participants prepared their gun in the gun preparation room and then received instructions either by the experimenter in the low stress (LS) scenario or by the role player in the high stress (HS) scenario. Participants were required to walk up the hallway taking self-protection measures. When they have reached the last door on the right side, they were required to open this door and search the room for the target person. As soon as the target person appeared in their visual field, they were required to fire six consecutive shots on the target person. Shooting distance was approximately 5 m. Target person was either represented by a mannequin in the LS scenario or a police instructor in the HS scenario. Scenarios were videotaped by two cameras.

The set-up of the scenarios is depicted in Figure 1. In both scenarios, participants were required to walk up a 20 m long hallway taking self-protection measures, until they reached the door of the last room on the right-hand side. All other rooms were marked and were not relevant in the scenario. As soon as participants had reached the last door, they had to open this door and search the room for the target person. Subsequently, participants had to fire six consecutive shots on the target person. In agreement with real-life situations and police guidelines, participants retreated behind the door and shot around the corner, resulting in a shooting distance of approximately 5 m. Recruits used a handgun that was identical to their duty weapon (Heckler & Koch, P30) but adjusted to shoot with colored soap cartridges (Simunition^®^, FX Marking Ammunition).

In the LS scenario, participants received precise instructions from the experimenter, eliminating ambiguity and uncertainty of the situation. Participants were instructed that they would encounter the target person in the last room and as soon as the target person would appear in their field of vision, they had to fire six consecutive shots. In the LS scenario, the target person was a life-sized mannequin that stood straight up, facing the participant and holding its arms as if pointing a gun toward the participant. The mannequin was supposed to eliminate the threat of physical harm. The mannequin was fitted with target areas indicating the score of a hit in this area. Participants were aware that shooting accuracy would be assessed but received no further explanations about the scoring grid.

In the HS scenario, participants received limited instructions from the experimenter increasing feelings of ambiguity, uncertainty and uncontrollability. They were told to act in accordance with the police guidelines in the following scenario and that they had to use their gun to solve the situation. Then, participants were sent toward the hallway – “the operation site” (see Figure 1) – where they encountered the civilian who called the police. This person was played by a male, Caucasian experimenter (32 years old). He suddenly came around the corner and approached the participant franticly: “Come quickly! There is a shooting! I heard shots in the last room! You need to go there!”. The involvement of the civilian is assumed to further increase psycho-social stress. In the HS scenario, the target person was an experienced police operation instructor who wore protective gear and a handgun loaded with colored soap cartridges. While the participants walked up the hallway, the instructor fired two or three shots, so that participants heard the shooting, which intended to induce physical threat. As soon as the participants opened the door, the instructor stood still in the same stance as the mannequin, pointing the gun at the participant. After the participants had fired six shots, the instructor played as being hurt and went down to the ground.

Both scenarios were timed using a digital stopwatch (CASIO, HS-3V-1RET). The scenarios started as soon as the police recruits entered the hallway leading up to the target room. After firing the last shot, the scenarios ended. On average, the LS scenario lasted 41 s (*SD* = 14) and the HS scenario 46 s (*SD* = 26). Additionally, both scenarios were videotaped with two cameras (GoPro HERO 4 Silver). One camera recorded the interaction of the participants with the experimenter/role player in the hallway, and the second camera recorded the shooting in the target room (see Figure 1). Videotapes were used for further analyses of shooting accuracy.

### Measures

#### Psychological Measures

Recruits’ state anxiety in response to the stress manipulation was measured during baseline measurements and directly after the scenarios using the Anxiety Thermometer (Houtman and Bakker, 1989). The Anxiety Thermometer is a 10-cm continuous scale on which participants rated their state anxiety they had experienced during the scenarios, ranging from *not anxious at all* to *extremely anxious*. Validity and test-retest reliability correlations coefficients range between 0.60 and 0.78 (Houtman and Bakker, 1989). For analyses, the entries were transformed into values ranging from 0 to 10.

Perceived mental effort was assessed during baseline measurements and directly after each scenario using the German version of the rating scale mental effort (RSME; Zijlstra, 1993; German version: Skala subjektiv erlebter Anstrengung, SEA; Eilers et al., 1986). The SEA is a continuous 110-mm scale, on which each 10 mm interval and verbal anchors are marked. Participants rated their perceived mental effort during the scenarios by marking the specific value on the scale. For analyses, entries were transformed into values ranging from 0 to 11.

Sensation seeking was measured using the German version of the Sensation Seeking Scale – Form V (SSS-V; Zuckerman, 1994; German version: Beauducel et al., 2003). Participants were required to answer either A or B for each of the 40 items. Answers A and B designate behaviors that are either characteristic of sensation seekers (“I would like to learn to fly an airplane”) or non-sensation seekers (“I would not like to learn to fly an airplane”). Total scores range between 0 and 40. The SSS-V has been used in over 600 studies and was found to have sufficient psychometric properties in many languages. Cronbach’s α in the norm sample was 0.82 (Beauducel et al., 2003) and in the present study 0.63. Recruits’ sensation seeking scores (*M* = 23.00, *SD* = 4.63, range = 15–31) were similar to the scores obtained by the norm sample (*M* = 22.70, *SD* = 5.70; Beauducel et al., 2003).

Dispositional self-control was assessed using the German version of the Self-Control Scale (SCS; Tangney et al., 2004; German version, SCS-K-D: Bertrams and Dickhäuser, 2009). It consists of 13 items which are answered on a 5-point Likert scale ranging from 1 = *not at all like me* to 5 = *very much like me*. Consequently, total scores range from 13 to 65. An example item is as follows: “People would say that I have iron self-discipline.” In the present study, the internal consistency of the SCS-K-D was satisfactory (α = 0.83) and similar to the one in the norm sample (α = 0.79). Overall, police recruits (*M* = 44.42, *SD* = 7.41) scored significantly above the norm sample, i.e., 39.85, *t*(18) = 2.69, *p* = 0.015, indicating that the recruits had considerably higher self-control than undergraduate psychology students tested by Tangney and Colleagues (2004).

#### Physiological Measures

**FIGURE 2:**
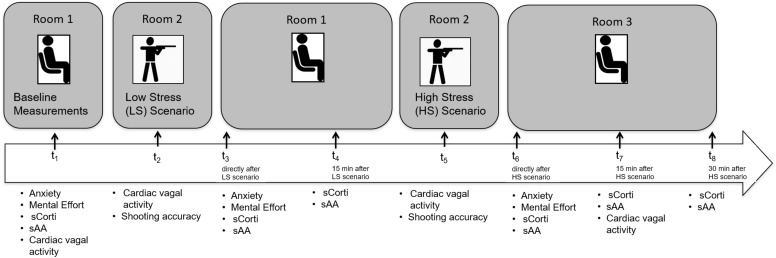
Overview of the study timeline. Participants were required to undergo two shooting scenarios in a low stress (LS) and high stress (HS) condition. Their psychological, physiological and behavioral responses were assessed at t_1_–t_8_. Anxiety and mental effort were assessed through questionnaires at three points. Salivary cortisol (sCorti) and salivary alpha-amylase (sAA) were assessed at six points. Cardiac vagal activity was recorded continuously, but four intervals were chosen for analyses. Dispositional self-control and sensation seeking were assessed through questionnaires at t_7_.

Salivary samples were collected using salivette sampling devices (Sarstedt AG & Co., Nümbrecht, Germany) at six points during the experiment (see Figure 2). Sampling time was exactly 1 min during which participants had to chew the cotton swabs as regularly as possible. Saliva samples were immediately stored in a coolbag with cooling elements. At the end of the testing day, they were stored at −20°C until further analyses. Biochemical analyses were conducted by the Steroid Laboratory of the Institute of Pharmacology, Heidelberg University, Germany. After thawing, saliva samples were centrifuged at 30000 rpm for 5 min, which resulted in a clear supernatant of low viscosity. Fifty microliters of saliva were used for duplicate analyses.

Free cortisol levels were measured using a commercially available immunoassay (IBL International, Hamburg, Germany). Intra- and interassay coefficients of variation were below 8% (Schultheiss and Stanton, 2009).

Salivary alpha-amylase levels were measured using the analyzer ADVIA Chemistry XPT (Siemens, München, Germany) and the reagents #03031177 (Siemens, München, Germany). Saliva was diluted 1:200 using 0.9% saline solution.

HRV as an index of cardiac vagal activity was measured using a wearable, portable, externally applied ECG recorder and wireless transmitter for ECG measurement (eMotion Faros 180°). Two disposable ECG pre-gelled electrodes (Ambu L-00-S/25, Ambu GmbH, Bad Nauheim, Germany) were placed below the right clavicle and on the left side of the chest below the 12th rib, respectively. Inter-beat intervals were continuously recorded with a sampling rate of 1000 Hz until 30 min after the start of the HS scenario. After sitting quietly for a few minutes, a baseline reading of cardiac vagal activity was recorded for 2 min. Since physical activity heavily influences HRV parameters, making the interpretation unambiguous, task cardiac vagal activity was assessed directly before the scenarios for 1 min following the recommendations (Esco and Flatt, 2014; Laborde et al., 2017). Post-task cardiac vagal activity was assessed 18 min after the start of the scenario for 2 min in a sitting position. HRV was analyzed using Kubios HRV (Biosignal Analysis and Medical Imaging Group, University of Eastern Finland, Finland). The full ECG recordings were inspected visually and artifacts were corrected manually (Laborde et al., 2017). Root Mean Square of Successive Differences (RMSSD) from time domain analyses was calculated for quantification of short-time HRV, as it is deemed a reliable measure of cardiac vagal activity under ambulatory trails since it is relatively free of respiratory influences as compared to high frequency parameters (Laborde et al., 2017). Cardiac vagal reactivity was calculated by subtracting baseline cardiac vagal activity from task cardiac vagal activity. Cardiac vagal recovery was calculated by subtracting task cardiac vagal activity from post-task cardiac vagal activity.

#### Behavioral Measure

Police recruits’ performance was assessed through shooting accuracy. Scores were assigned to hits on different body parts of the opponent (hips: 50, chest: 20, head: 20, legs: 10, arms: 5), representing the effectiveness of a hit in this area (as rated by an experienced police instructor). In line with the current police guidelines, hits in the hips were awarded with the greatest scores. Hits in the respective areas were identified by the police instructor and a research assistant with the help of the video material. Shooting accuracy was then calculated by sum of the scores divided by maximum score (maximum score = number of shots ^*^ 50).

### Procedure

All testing sessions were conducted during regular training sessions between 02.00 p.m. and 08.00 p.m., when cortisol levels are most stable (Kudielka et al., 2004). The sessions took place in a vacant office building that serves as a police training location. Participants were instructed to refrain from smoking, eating, or drinking any beverages except water at least 1 h prior to the study and during the study protocol. An overview of the study timeline is displayed in Figure 2. After participation, participants were fully debriefed about the aims of the study and were instructed not to talk to each other about the content of the study.

### Statistical Analyses

All dependent variables were checked for outliers (three interquartile ranges above 3rd/under 1st quartile; Tukey, 1977) and normal distribution was tested using the Kolmogorov–Smirnov test. According to the recommendations of Kirschbaum (1991), sCorti values and the variable of cardiac vagal activity were naturally log-transformed.

To investigate the effect of the stress manipulation on anxiety, mental effort, HRV, sCorti, sAA and shooting accuracy in the HS and LS scenarios were compared using repeated measures ANOVAs and contrasts (difference). For psychological variables a 1 × 3 ANOVA (time: t_1_, t_3_, t_6_) was conducted, for sCorti and sAA a 1 × 6 ANOVA (time: t_1_, t_3_, t_4_, t_6_, t_7_, t_8_), for cardiac vagal activity a 1 × 4 ANOVA (time: t_1_, t_2_, t_5_, t_7_) and for the behavioral variable a 1 × 2 ANOVA (LS vs. HS scenario). Greenhouse–Geisser corrected *p*-values were reported when the assumption of sphericity was violated as indicated by the Mauchly test. Significant main effects were further analyzed with Bonferroni corrected *post hoc* tests. To explore the contribution of coping-related variables to the stress responses and shooting accuracy, bivariate correlations were run, followed by hierarchical stepwise linear regression analyses. Using hierarchical regression, anxiety, mental effort, sCorti, sAA, cardiac vagal activity, cardiac vagal reactivity, cardiac vagal recovery and shooting accuracy, respectively in the LS and HS scenario, were entered as dependent variables. The first block included dispositional self-control, sensation seeking, and baseline cardiac vagal activity. The second block was used to explore the contribution of the situational stress responses. For cardiac vagal reactivity and recovery, task and post-task cardiac vagal activity were excluded at this stage, as reactivity and recovery are derived from these tonic variables.

All statistical analyses were performed using IBM SPSS 21 (Chicago, IL, United States). *p*-values < 0.05 are considered significant and for ANOVAs, $\eta_{p}^{2}$ was presented as a measure of effect size. In this regard, values ≈0.02 represent a small effects, values ≈0.15 represent medium-size effects and values ≈0.35 represent large effects (Cohen, 1992).

## Results

Outlier analysis revealed two outliers in anxiety at t_1_ and three outliers in cardiac vagal activity (at t_1_, t_4_, and t_7_). Normal distribution was violated in three variables. As ANOVAs are robust against this violation, variables were not transformed (Schmider et al., 2010).

### Differences Between Low and High Stress Scenarios

Descriptive data of anxiety, mental effort, cardiac vagal activity, sCorti, sAA, and shooting accuracy are presented in Table 1.

**TABLE 1:** Descriptive statistics of anxiety, mental effort, sCorti, sAA, cardiac vagal activity, and shooting accuracy at each measurement point.

		***M***	***SD***
Anxiety (*N* = 19)	t_1_	1.92	1.55
	t_3_	4.63	2.25
	t_6_	6.46	1.71
Mental effort (*n* = 17)	t_1_	1.76	1.72
	t_3_	3.71	2.38
	t_6_	6.12	2.35
sCorti in nmol/l (*n* = 17)	t_1_	4.59	2.84
	t_3_	5.00	2.66
	t_4_	6.53	4.13
	t_6_	5.94	3.21
	t_7_	5.61	3.94
	t_8_	4.56	2.91
sAA in U/ml (*N* = 19)	t_1_	154.57	88.49
	t_3_	168.73	98.48
	t_4_	121.57	68.14
	t_6_	156.51	82.38
	t_7_	124.52	77.02
	t_8_	100.87	55.33
Cardiac vagal activity in ms (*n* = 14)	t_1_	57.41	41.98
	t_2_	34.15	39.16
	t_5_	28.18	24.82
	t_7_	57.04	63.48
Shooting accuracy (*n* = 16)	LS scenario	22.66	9.52
	HS scenario	24.39	12.43

*HR, heart rate; sCorti, salivary cortisol; sAA, salivary alpha-amylase; LS, low stress; HS, high stress.*

#### Psychological Stress Responses

**FIGURE 3:**
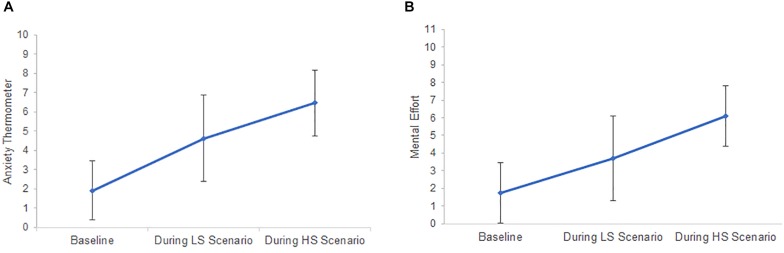
Psychological response profiles to the low stress (LS) and high stress (HS) scenario regarding mean anxiety **(A)** and mean mental effort **(B)**. Error bars represent standard deviation.

Anxiety and mental effort during the experiment are depicted in Figure 3.

Anxiety changed significantly over time, *F*(2,36) = 39.90, *p* < 0.001, $\eta_{p}^{2}$ = 0.69. Contrasts show that anxiety significantly increased from baseline in the LS scenario [*F*(1,18) = 24.04, *p* < 0.001, $\eta_{p}^{2}$ = 0.57] and further increased in the HS scenario, *F*(1,18) = 61.95, *p* < 0.001, $\eta_{p}^{2}$ = 0.76. All pairwise comparisons yielded significant results (all *p* < 0.006).

Similarly, the stress manipulation affected the reported mental effort, *F*(2,32) = 31.88, *p* < 0.001, $\eta_{p}^{2}$ = 0.67. Participants invested more mental effort in the LS scenario than during baseline [*F*(1,16) = 10.92, *p* = 0.004, $\eta_{p}^{2}$ = 0.41] and more mental effort in the HS than in the LS scenario, *F*(1,16) = 60.66, *p* < 0.001, $\eta_{p}^{2}$ = 0.79. Again, all *post hoc* pairwise comparisons yielded significant results (all *p* < 0.013).

#### Physiological Stress Responses

**FIGURE 4:**
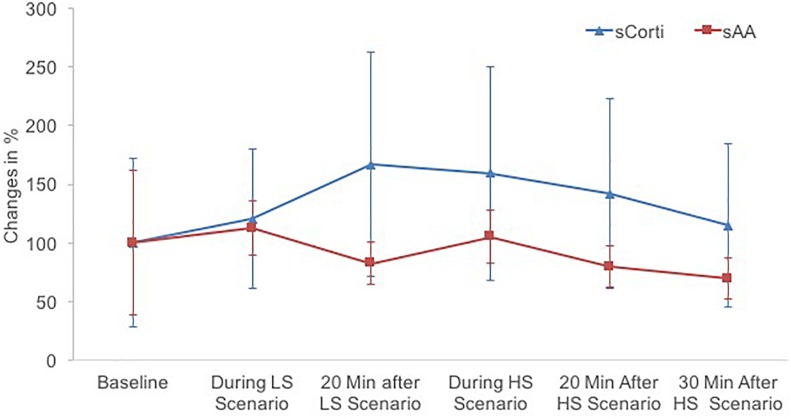
Physiological response profiles to the low stress (LS) and high stress (HS) scenario regarding mean salivary cortisol (sCorti) and mean salivary alpha-amylase (sAA). Error bars represent standard deviation.

**FIGURE 5:**
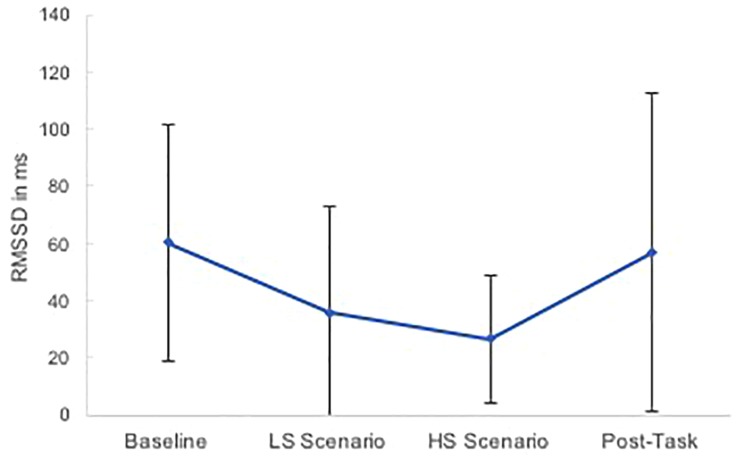
Cardiac vagal response profiles to the low stress (LS) and high stress (HS) scenario. Error bars represent standard deviation.

Changes in sCorti, sAA and HRV in the course of the experiment are depicted in Figures 4, 5.

sCorti levels significantly varied in the course of the experiment, *F*(2.02,32.34) = 4.36, *p* = 0.021, $\eta_{p}^{2}$ = 0.21. Results of the contrast analysis showed that sCorti levels significantly raised after the LS scenario, *F*(1,16) = 7.01, *p* = 0.018, $\eta_{p}^{2}$ = 0.31. However, they did not further increase after the HS scenario, *F*(1,16) = 0.02, *p* = 0.891, $\eta_{p}^{2}$ < 0.01. In contrast, Bonferroni corrected *post hoc* tests demonstrated that sCorti levels significantly decreased after the LS scenario (from t_4_, t_6_ and t_7_ to t_8_, all *p* < 0.021), but baseline sCorti levels at t_1_ and t_3_ did not significantly differ from sCorti levels at any other measurement point (all *p* > 0.237).

Stress manipulation had a significant impact on sAA levels, *F*(2.90,52.13) = 16.47, *p* < 0.001, $\eta_{p}^{2}$ = 0.48. Contrast analysis showed that sAA did not significantly increase from baseline in the LS scenario [*F*(1,18) = 2.45, *p* = 0.135, $\eta_{p}^{2}$ = 0.12] and from t_4_ in the HS scenario, *F*(1,18) = 3.09, *p* = 0.096, $\eta_{p}^{2}$ = 0.15. Bonferroni corrected post-hoc tests revealed that baseline sAA values were relatively high, as they differed significantly from the values in other resting conditions (i.e., t_4_, t_7_, and t_8_; all *p* < 0.026). sAA values after the LS and HS scenario were significantly higher than values in the resting conditions other than baseline (all *p* < 0.031), which did not significantly differ from each other (all *p* > 0.119).

Cardiac vagal activity significantly varied in the course of the experiment, *F*(3,39) = 9.79, *p* < 0.001, $\eta_{p}^{2}$ = 0.43. Results of the contrast analysis showed that cardiac vagal activity significantly decreased from baseline to the LS scenario [*F*(1,13) = 12.72, *p* = 0.003, $\eta_{p}^{2}$ = 0.50] and further decreased to the HS scenario, *F*(1,13) = 13.48, *p* = 0.003, $\eta_{p}^{2}$ = 0.51. After the HS scenario, cardiac vagal activity increased significantly, *F*(1,13) = 4.77, *p* = 0.048, $\eta_{p}^{2}$ = 0.27. Bonferroni-corrected *post hoc* tests supported that cardiac vagal activity significantly decreased from baseline to the LS scenario (*p* = 0.021) and to the HS scenario (*p* = 0.006). However, cardiac vagal activity in the LS and HS scenario as well as during baseline and post-task did not significantly differ from each other (all *p* = 1).

#### Behavioral Stress Response

**FIGURE 6:**
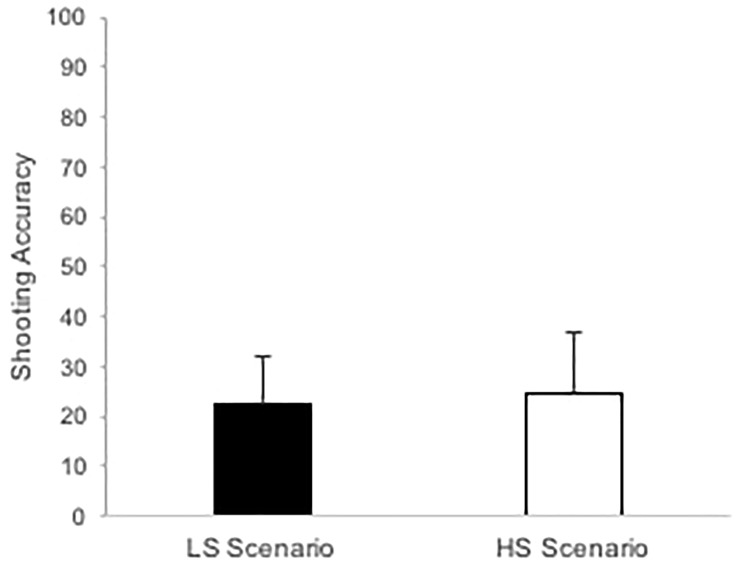
Mean percentage of shooting accuracy in the low stress (LS) and high stress (HS) scenario. Error bars represent standard deviation.

Shooting accuracy in the LS vs. HS scenario is depicted in Figure 6. Shooting accuracy did not differ between the LS and HS scenario, *F*(1,15) = 0.14, *p* = 0.718, $\eta_{p}^{2}$ = 0.01.

### Correlations

Tables 2, 3 show an overview of Pearson’s correlation coefficients of all study variables in the LS and HS scenario, respectively.

**TABLE 2:** Correlation matrix for low stress (LS) scenario.

	**1**	**2**	**3**	**4**	**5**	**6**	**7**	**8**	**9**	**10**	**11**	**12**
(1) Self-control	—	–0.26	–0.62^∗∗^	−0.54^*^	0.30	0.06	0.23	0.17	–0.03	–0.52	–0.11	–0.32
(2) Sensation Seeking		—	0.30	0.17	0.35	–0.38	–0.18	0.13	–0.04	0.09	0.10	0.12
(3) Anxiety			—	0.79^∗∗∗^	–0.18	0.29	−0.50^*^	–0.31	–0.45	0.39	–0.17	0.05
(4) Mental Effort				—	–0.18	0.37	–0.33	–0.19	−0.51^*^	0.22	–0.39	–0.36
(5) sCorti (20 min after LS scenario)					—	–0.03	–0.31	–0.31	–0.26	–0.11	0.04	0.02
(6) sAA						—	–0.01	–0.04	–0.15	0.31	–0.29	–0.15
(7) Baseline CVA							—	0.55^*^	0.56^*^	–0.48	–0.03	–0.21
(8) Task CVA								—	0.57^*^	0.30	–0.14	–0.01
(9) Post-task CVA									—	0.14	0.67^∗∗^	0.21
(10) Reactivity CVA										—	–0.06	0.33
(11) Recovery CVA											—	0.17
(12) Shooting accuracy												—

sCorti, salivary cortisol; sAA, salivary alpha-amylase; CVA, cardiac vagal activity; ^*^p < 0.05, ^∗∗^*p* < 0.01, ^∗∗∗^*p* < 0.001.

**TABLE 3:** Correlation matrix for high stress (HS) scenario.

	**1**	**2**	**3**	**4**	**5**	**6**	**7**	**8**	**9**	**10**	**11**	**12**
(1) Self-control	—	–0.26	−0.51^*^	–0.25	0.14	–0.01	0.23	0.18	–0.02	−0.55^*^	–0.03	–0.00
(2) Sensation Seeking		—	–0.10	–0.29	0.29	–0.27	–0.18	–0.10	–0.04	0.18	0.08	–0.30
(3) Anxiety			—	0.64^∗∗^	–0.12	0.19	–0.07	0.02	0.09	0.40	–0.05	0.10
(4) Mental Effort				—	–0.16	0.59^∗∗^	0.01	0.35	–0.25	0.29	−0.54^*^	0.08
(5) sCorti (20 min after HS scenario)					—	–0.03	–0.47	–0.33	–0.41	0.26	–0.12	–0.03
(6) sAA						—	0.01	0.12	–0.17	0.04	–0.20	–0.10
(7) Baseline CVA							—	0.50	0.56^*^	–0.88^∗∗∗^	0.16	0.02
(8) Task CVA								—	0.53^*^	–0.14	0.07	–0.32
(9) Post-task CVA									—	–0.28	0.84^∗∗∗^	–0.34
(10) Reactivity CVA										—	–0.13	–0.13
(11) Recovery CVA											—	–0.31
(12) Shooting accuracy												—

sCorti, salivary cortisol; sAA, salivary alpha-amylase; CVA, cardiac vagal activity; ^*^p < 0.05, ^∗∗^*p* < 0.01, ^∗∗∗^*p* < 0.001.

Dispositional self-control correlated significantly with anxiety in the LS and HS scenario (negatively) and with cardiac vagal reactivity in the HS scenario (negatively). Cardiac vagal activity at baseline correlated significantly with anxiety in the LS scenario (negatively). Post-task cardiac vagal activity correlated significantly with mental effort in the LS scenario (negatively). Similarly, cardiac vagal recovery correlated significantly with mental effort in the HS scenario (negatively).

### Regression Analyses

Hierarchical stepwise regressions were performed to predict psychophysiological stress responses (i.e., anxiety, mental effort, sCorti, sAA, cardiac vagal activity, cardiac vagal reactivity, cardiac vagal recovery) and performance (i.e., shooting accuracy) in the LS and HS scenario, respectively. The first block was used to identify salient dispositional predictors (sensation seeking, self-control, and baseline cardiac vagal activity) and the second block included the remaining stress responses (i.e., anxiety, mental effort, sCorti 20 min after the scenario, sAA directly after the scenario, task cardiac vagal activity, post-task cardiac vagal activity, cardiac vagal reactivity, and cardiac vagal recovery). Regressions can be found in Tables 4, 5.

**TABLE 4:** Multiple (stepwise) regressions for stress responses in the low stress (LS) scenario.

	**Unstandardized coefficients**		**Standardized coefficients**	
		
**Model**	***B***	***SE***	**β**	***T***
**Shooting accuracy**				
(1) Self-control	−1.69	0.59	−0.69	−2.85^*^
**Anxiety**				
(1) Self-control	−0.26	0.07	−0.76	−3.83^*^
(2) Self-control	−0.16	0.06	−0.46	−2.58^*^
Mental Effort	0.49	0.16	−0.55	3.11^*^
**Mental Effort**				
(1) Anxiety	0.90	0.20	0.80	4.39^∗∗^
**sAA**				
(1) CVA recovery	−42.92	19.07	−0.56	−2.25^*^
**Cardiac vagal activity**				
(1) CVA baseline	0.99	0.45	0.56	2,22^*^
**Cardiac vagal recovery**				
(1) sAA	−0.01	0.00	−0.56	−2.25^*^

sCorti, salivary cortisol; sAA, salivary alpha-amylase; CVA, cardiac vagal activity; ^*^p < 0.05, ^∗∗^*p* < 0.01.

**TABLE 5:** Multiple (stepwise) regressions for stress responses in the high stress (HS) scenario.

	**Unstandardized coefficients**		**Standardized coefficients**	
		
**Model**	***B***	***SE***	**β**	***T***
**Shooting accuracy**				
(1) Post-task CVA	−9.98	4.17	−0.62	−2.40^*^
**Anxiety**				
(1) Mental effort	0.37	0.15	0.62	2.49^*^
**Mental effort**				
(1) Anxiety	1.02	0.41	0.62	2.49^*^
(2) Anxiety	1.07	0.31	0.65	3.41^∗∗^
CVA recovery	−1.93	0.67	−0.54	−2.86^*^
(3) Anxiety	0.90	0.25	0.55	3.67^∗∗^
CVA recovery	−1.84	0.51	−0.52	−3.58^∗∗^
sAA	0.01	0.01	0.41	2.75^*^
(4) Anxiety	0.86	0.18	0.52	4.70^∗∗^
CVA recovery	−1.89	0.38	−0.53	−4.98^∗∗^
sAA	0.01	0.00	0.36	3.21^*^
Task CVA	0.85	0.31	0.31	2.77^*^
**Cardiac vagal reactivity**				
(1) Baseline CVA	−1.19	0.20	−0.88	−5.99^∗∗∗^
(2) Baseline CVA	−0.80	0.20	−0.59	−3.93^∗∗^
Self-control	−0.06	0.02	−0.43	−2.87^*^
(3) Baseline CVA	−0.97	0.18	−0.73	−5.56^∗∗^
Self-control	−0.06	0.02	−0.40	−3.33^∗∗^
sCorti	−0.27	0.11	−0.25	−2.52^*^

sCorti, salivary cortisol; sAA, salivary alpha-amylase; CVA, cardiac vagal activity; ^*^p < 0.05, ^∗∗^*p* < 0.01, ^∗∗∗^*p* < 0.001.

#### Regression Analyses in the LS Scenario

For shooting accuracy, the regression analysis identified self-control as a significant predictor, *R*^2^ change = 0.48, *p* = 0.019. It accounted for 48% (adjusted *R*^2^ = 0.42) of the variance in shooting accuracy in the LS scenario. For anxiety, regression analyses identified two significant predictors. Self-control was extracted as the first factor, *R*^2^ change = 0.57, *p* = 0.003. The second factor was mental effort, *R*^2^ change = 0.21, *p* = 0.011. Both predictors accounted for 78% (adjusted *R*^2^ = 0.74) of the variance in anxiety in the LS scenario. For mental effort, anxiety was identified as significant predictor (*R*^2^ change = 0.64, *p* = 0.001), accounting for 64% (adjusted *R*^2^ = 0.60) of the variance. In the LS scenario, sAA was significantly predicted by cardiac vagal recovery (*R*^2^ change = 0.32, *p* = 0.046) explaining 32% (adjusted *R*^2^ = 0.25) of the variance in the LS scenario. Cardiac vagal activity in the LS scenario was significantly predicted by cardiac vagal activity at rest, *R*^2^ change = 0.31, *p* = 0.048 accounting for 31% (adjusted *R*^2^ = 0.25) of variance. For cardiac vagal recovery, sAA was extracted as a significant factor, *R*^2^ change = 0.32, *p* = 0.046. It accounted for 32% (adjusted *R*^2^ = 0.25) of variance.

For sCorti and cardiac vagal reactivity in the LS scenario, no significant predictors were extracted.

#### Regression Analyses in the HS Scenario

For shooting accuracy, post-task cardiac vagal activity was identified as a significant predictor, *R*^2^ change = 0.39, *p* = 0.040, explaining 39% (adjusted *R*^2^ = 0.32) of the variance in the HS scenario. Anxiety in the HS scenario was significantly predicted by mental effort, *R*^2^ change = 0.38, *p* = 0.032, accounting for 38% (adjusted *R*^2^ = 0.32) of the variance. For mental effort in the HS scenario, four factors were extracted. The first factor identified was anxiety (*R*^2^ change = 0.38, *p* = 0.032), the second factor was cardiac vagal recovery (*R*^2^ change = 0.29, *p* = 0.019), the third factor was sAA (*R*^2^ change = 0.16, *p* = 0.025) and the fourth was cardiac vagal activity (*R*^2^ change = 0.09, *p* = 0.028). All predictors together explained 92% (adjusted *R*^2^ = 0.88) of the variance of mental effort in the HS scenario. Cardiac vagal reactivity in the HS scenario was significantly predicted by three factors. The first predictor identified was baseline cardiac vagal activity (*R*^2^ change = 0.78, *p* < 0.001), the second predictor was self-control (*R*^2^ change = 0.10, *p* < 0.018) and the third predictor was sCorti 20 min after the scenario (*R*^2^ change = 0.05, *p* = 0.036). Together, they accounted for 94% (adjusted *R*^2^ = 0.91) of the variance.

For sCorti, sAA and cardiac vagal activity in the HS scenario, no significant predictors were extracted.

## Discussion

The present study investigated the psychological (i.e., anxiety, mental effort), physiological (i.e., sCorti, sAA, cardiac vagal activity) and behavioral responses (i.e., shooting accuracy) of police recruits during a simulated shooting scenario under a HS and a LS condition. The aim of the study was twofold: (1) to examine the stress responses of police recruits in reality-based scenarios and (2) to examine the effects of stress responses and coping-related personality traits (i.e., sensation seeking and self-control) on shooting performance.

Hypothesis 1 predicted elevated psychophysiological stress responses in the HS scenario as compared to the LS scenario. It was supported by the psychological, but not by the physiological stress responses. Police recruits reported significantly higher levels of anxiety and mental effort in the HS scenario as compared to the LS scenario. This finding is in line with other studies that incorporated LS vs. HS conditions for police officers during handgun shooting or similar situations (Nieuwenhuys and Oudejans, 2010, 2011; Taverniers and De Boeck, 2014). In contrast, recruits showed similar physiological stress reactions to the LS and HS scenario. That is, sAA significantly increased and cardiac vagal activity significantly decreased during both scenarios compared to baseline conditions. While the increases in sAA and decreases in cardiac vagal activity during reality-based scenarios complement a great body of research showing strong physiological stress responses to reality-based scenarios (Groer et al., 2010; Taverniers and De Boeck, 2014; Brisinda et al., 2015; Strahler and Ziegert, 2015), the missing differentiation between the LS and HS scenario might be explained through the nature of the scenarios. Brisinda et al. (2015) were able to distinguish medium- und high-risk scenarios based on cardiac vagal activity patterns. However, in their study, only high-risk scenarios, but not medium-risk scenarios involved shooting tasks, whereas in the current study, even the LS scenario required shooting. sCorti followed an unexpected pattern with the highest levels 20 min after the LS scenario and thereafter, steadily decreasing, which is comparable to findings of decreasing cortisol after simulated police incidents (Strahler and Ziegert, 2015; Arble et al., 2019). Strahler and Ziegert (2015) suggested blunted cortisol responses due to long-term chronic stress as well as adaption to endocrine stress responses following frequent encounters of acute stress as possible explanations for this finding. In contrast, the missing profound sCorti response after the HS scenario contradicts the findings of increased sCorti levels after reality-based scenarios in police officers (Regehr et al., 2008; Groer et al., 2010). Possibly, police recruits’ sCorti levels did not further increase after the HS scenario because they were still in the midst of dealing with the first acute stress response to the LS scenario.

Subsequent to the inconsistent pattern of psychological and physiological stress responses, hypothesis 2, predicting decreases in shooting accuracy in the HS scenario, was not supported. Shooting accuracy in the LS and HS scenario was extremely low and did not differ significantly from each other. Nonetheless, in line with the attentional control theory (Eysenck et al., 2007), efficiency decreased in the HS scenario, since police recruits reported to invest more mental effort in the HS scenario. Others found decreased shooting performance under HS compared to LS conditions (Nieuwenhuys and Oudejans, 2010, 2011; Taverniers and De Boeck, 2014) but the divergent pattern in our study might be due to near floor performance under LS and/or the elevated physiological stress responses in the LS scenario.

Contradicting hypothesis 3, neither baseline cardiac vagal activity nor cardiac vagal reactivity predicted shooting performance in the LS and HS scenario. However, shooting performance was significantly predicted by post-task cardiac vagal activity in the HS scenario. Surprisingly, higher levels of post-task cardiac vagal activity were associated with worse shooting performance. Given the correlation between post-task and task cardiac vagal activity (see Table 3), police recruits with high levels of cardiac vagal activity in recovery might also have high levels of cardiac vagal activity during the task. In a previous study on shooting performance under stress, a larger vagal withdrawal in the HS, but not in the LS condition, was found to be adaptive (Mosley et al., 2017). Similarly, the high levels of post-task cardiac vagal activity in the current study might reflect a smaller cardiac vagal withdrawal during the task, resulting in worse shooting performance, as police recruits might have not met the high situational demands of the HS scenario (Mosley et al., 2017). As findings on the link between cardiac vagal reactivity and performance under stress are diverse, further research is needed to clarify the role of cardiac vagal activity as an indicator of self-regulation under differing situational demands.

As a coping-related personality trait, sensation seeking did not predict shooting performance nor stress responses in the LS and HS scenario. This finding does not support hypothesis 4 and contradicts a body of research suggesting stress-buffering effects of sensation seeking (Couture et al., 2008; Tschiesner, 2012; Frenkel et al., 2018; Frenkel et al., unpublished). Therefore, further investigations into the role of sensation seeking are necessary to allow deeper conclusions. Previous studies with significant findings on sensation seeking made use of dichotomization into extreme groups of low and high sensation seekers (Shabani et al., 2011; Frenkel et al., 2018). In future studies with police officers, the comparison of low and high sensation seekers might increase effect sizes and reveal the hypothesized differences in stress responses. Furthermore, it might be especially interesting to investigate samples of experienced police officers or special forces, in which a self-selection based on relevant personality traits might have occurred. The current sample consisted of relatively unexperienced police recruits. Work experience has been identified as a predictor for less anxiety and better performance under stressful circumstances (Vickers and Lewinski, 2012; Landman et al., 2016). Therefore, more experienced police officers and special forces might yield different results.

Hypothesis 5, predicting that self-control positively influences shooting performance, was not supported. In the LS scenario, self-control significantly predicted anxiety and shooting performance: While higher self-control was associated with less anxiety, higher self-control also worsened shooting performance. In contrast, high levels of self-control were associated with less cardiac vagal reactivity in the HS scenario, which was assumed to be beneficial for shooting performance. Landman et al. (2016) found that self-control reduced the impact of stress on shooting accuracy, but not on anxiety and HR. This finding suggests that self-control helps police officers to prevent psychophysiological stress responses from influencing their attention and behavior, without reducing the emotional response itself. In line with mindfulness- and acceptance-based theory (Gardner and Moore, 2004), they argued that attempting to suppress or change the unwanted thoughts and emotions might even be counter-productive as it might decrease goal-directed attention. This explanation is supported by the negative association of trait emotionality and shooting performance of rifle shooters (Mosley et al., 2018): the ability to ignore the emotional state during shooting in HS circumstances improved shooting performance. Possibly, in the current study, police recruits with high levels of self-control tried to regulate their anxiety in the LS scenario, which decreased their goal-direct attention resulting in inaccurate shooting performance. These findings across several studies further strengthen the need for future research that integrates several coping-related variables in order to gain a more holistic view of performance under differing situational demands.

To date, there are only few studies that integrate coping-related variables, endocrine, autonomic and emotional stress responses as well as occupationally meaningful behavior to investigate performance under stress (Baumeister et al., 2007; Driskell et al., 2008). Applying a reality-based environmental stressor of high external validity is a feasible way to mimic stressful real-life settings. So far, previous studies comparing LS vs. HS conditions usually used artificial, static shooting tasks in the LS condition and only implemented complex scenarios in the HS condition (Taverniers and De Boeck, 2014). Therefore, the present study was the first attempt to create two comparable naturalistic shooting scenarios of varying stress intensities. Various stressors that had been identified to be relevant for police work (i.e., uncertainty, physical threat and social evaluation) have been integrated in the HS scenario used in the present study. However, it is striking that even in the LS scenario, which lacked these stressors, physiological stress responses were high while shooting accuracy was low. This finding is in line with the claim that police training does not appear to sufficiently prepare police officers for the demands on duty (Morrison and Vila, 1998) and that the officers themselves demand more realistic training conditions (Renden et al., 2015). Currently, training of police officers primarily focuses on equipping them with technical, physical and tactical skills, while emotional and psychological components of performance are neglected. As a result, many training practices do not adequately simulate the conditions likely to be experienced by officers in real-world encounters (Morrison and Vila, 1998; Andersen et al., 2016b). The results of the present study highlight that police training should consist of high-realism scenarios that provide police officers with the opportunity to experience how psychological and physiological arousal impacts their behavior under stress and afford an opportunity to improve performance under extreme stress. Integrating psychological skills training into standard training curricula might increase positive outcomes in both acute stress situations and in health-related, long-term consequences (Papazoglou and Andersen, 2014). Research on police training has shown that training with threat-induced anxiety (e.g., through opponents shooting back) improves perceptual motor performance under stress (Oudejans, 2008; Nieuwenhuys and Oudejans, 2011; but see Nieuwenhuys et al., 2015). However, these training interventions have not specifically addressed dealing with unwanted thoughts and emotions. Therefore, psychoeducation during training should emphasize the adaptive function of psychophysiological stress responses enhancing job performance in acute stress situations and also explain the negative long-term effects of chronic stress responses on physical and mental health (Papazoglou and Andersen, 2014). Given the results on self-control, for future effective police training, it might be interesting to combine training under stress with techniques aiming at accepting psychophysiological arousal and maintaining a goal-directed focus (Christopher et al., 2018). Confronting officers with their own psychophysiological stress responses (e.g., using inexpensive heart rate monitors) during realistic scenario training might make them aware of the importance of managing stress responses as a benefit to their health (Papazoglou and Andersen, 2014).

There are also some limitations that need to be considered in light of the current study. Notably, generalizability is limited due to the small sample of police recruits which was sufficient to detect some hypothesized effects, but regression analyses might have been underpowered. Although police recruits were licensed to carry a gun and had delivered police services on duty already, they might have yielded different results than sworn police officers. Professional experience has been identified as a moderator of psychophysiological stress responses (McEwen, 2008; Landman et al., 2016; but see Strahler and Ziegert, 2015) and shooting performance under stress (Vickers and Lewinski, 2012). However, overall, sworn police officers show similar patterns of elevated psychophysiological stress responses and impaired performance in critical incidents (Nieuwenhuys and Oudejans, 2010, 2011; Taverniers and De Boeck, 2014). Clearly, the difference between novice and expert police officers warrants a larger follow-up study to clarify the role of work experience in stress responses and performance under stress.

Overall sCorti and sAA levels in the present study are relatively low (for comparison see Taverniers and De Boeck, 2014; Strahler and Ziegert, 2015). Although the mean increase of 1.94 nmol/l from baseline to 20 min after the LS scenario equals the increase after a reality-based scenario observed by Groer et al. (2010; *M* = 1.93), the missing profound sCorti reaction may be explained by the short duration of the scenarios. Groer et al. (2010) found markedly increased sAA and sCorti levels after a lengthy scenario of 6 min duration, while sCorti was not increased after a shorter scenario of 2 min duration. Similarly, our results also suggest that sAA is a more sensitive biomarker of acute stress events of short duration than sCorti.

We consider the reality-like environment in the present scenarios a strength, but it has the potential drawback that experimental control was limited. While increased anxiety in the HS scenario hints at higher perceived stress levels in the HS scenario, the physiological stress responses suggest similar stress responses for both scenarios, diminishing potential behavioral differences between the LS and HS scenario. This discrepancy between psychological and physiological aspects of the stress response is an often-reported phenomenon, whereby the underlying mechanisms are still not completely understood (e.g., Campbell and Ehlert, 2012). Based on previous literature on the nature of stressors, the manipulation of stress intensity should have worked fine, but the fixed order of conditions might have caused physiological stress levels in the LS scenario to be elevated and stress levels in the HS scenario to be lowered, respectively. Learning experiences in the LS scenario might have improved performance in the HS scenario: First, experiences in the LS scenario might have directly improved behavior because of knowledge or skill acquisition during the LS scenario. Second, positive experiences in the LS scenario might have changed cognitive appraisal processes concerning the upcoming HS scenario (Lazarus and Folkman, 1984). High sAA baseline levels hint at anticipatory stress before the LS scenario. Given that the scenarios might have been too short to activate the HPA axis, the high sCorti levels 20 min after the LS scenario could be interpreted as an additional indicator of anticipatory stress. The police recruits, who were relatively unexperienced in shooting scenarios, might have expected extremely stressful scenarios that succeed their coping resources, resulting in elevated stress levels (Lazarus and Folkman, 1984). Engaging in trained routines and mastering the LS scenario, police recruits might have experienced that their coping resources are sufficient to meet the demands of the scenario, which might have in turn reduced their stress response (Lazarus and Folkman, 1984). This interpretation is in line with several findings of physiological indicators of police officers’ anticipatory stress at the start of each shift (Anderson et al., 2002; Zefferino et al., 2006) and before amok training (Strahler and Ziegert, 2015). Further research is necessary to determine which aspects of reality-based scenarios exactly cause increased anxiety and physiological stress responses. Certainly, novelty, unpredictability and threat to physical integrity applied in this study are valid options to test in future research. For these studies, it might be beneficial to use virtual reality, as it can be designed and manipulated to experience many different conditions (Düking et al., 2018). Combined with real-time assessment of physiological parameters (e.g., HRV, see Brisinda et al., 2015), it allows to directly link a stress response to a presented stress cue.

Furthermore, the operationalization of shooting accuracy must be critically discussed. Integrating a score that reflects the effectiveness of a hit in a specific part of the body makes the measure of shooting accuracy more sensitive to changes in shooting accuracy. In line with current police guidelines of shooting behavior, the highest score (score = 50) was attached to the hip, as police officers are required to aim for the hip. However, no explicit instructions about the scores attached to each target area were provided in the present study. Most of the police recruits appeared to aim for the chest (score = 20), as a hit in this area is highly effective and offers a larger target area than the hip. In this sense, aiming for the chest might be highly adaptive, but was not rewarded in the present study. However, *post hoc* analyses with a simplified scoring grid (considering all hits on either hip, chest or head as an hit) also demonstrated no significant difference between the LS and HS scenario.

Additionally, previous studies suggest that psychophysiological stress responses might have differential effects on police work performance (Renden et al., 2014, 2017; Arble et al., 2019). While observational studies report a negative effect of stress on tactical behaviors such as handcuffing or arrest procedures (Renden et al., 2014), increases in physiological stress responses did not impair general police work performance, but specifically impaired verbal communication as compared to tactical performance and non-verbal communication (Arble et al., 2019). These differential effects demonstrate the complex nature of effective police work and the difficulties to define and asses ideal police performance. Future studies should apply reality-based scenarios that allow performance measurements of all relevant police skill domains to gain a holistic view of police work performance under stress.

The present study raises several issues that require future research. It seems well-documented that police officers experience severe psychophysiological stress responses (Groer et al., 2010; Taverniers and De Boeck, 2014; Strahler and Ziegert, 2015; Arble et al., 2019) and show impaired perceptual-motor performance under stress (Nieuwenhuys and Oudejans, 2010, 2011, 2017; Renden et al., 2014, 2017; Arble et al., 2019). However, it remains unclear through which mechanisms performance can be maintained. In the attentional control theory, individuals are assumed to spend extra mental effort in an attempt to lower anxiety, inhibit stimulus-driven impulses and/or enforce goal-directed control (Nieuwenhuys and Oudejans, 2017). So far, no study has directly tested how mental effort needs to be utilized to effectively maintain performance. Findings of the current study as well as of Landman et al. (2016) point in the direction that mental effort should not be spent on down-regulating anxiety. In contrast, a collection of experiments suggests a crucial role of inhibitory functions for performance under stress (Ducrocq et al., 2016). When elucidating the underlying mechanisms of mental effort, the role of psychophysiological stress responses should be considered: Do psychophysiological stress responses need to be decreased *per se* to maintain performance? Or do self-regulatory processes prevent stress responses from negatively influencing attention and performance? Understanding the mechanisms of how performance can be maintained under stress will allow the conceptualization of effective police training interventions.

Considering the health-related consequences of constant stress experiences, more research is needed to investigate how chronic stressors (e.g., shift work, work overload, fear of danger; Anderson et al., 2002) and/or frequent exposure to critical incidents adds up to allostatic load or overload in police officers and how this affects work performance under acute stress (McEwen, 1998). Therefore, future studies should expose police officers to multiple stressors limiting the availability of energetic resources (e.g., due to preceding physical exhaustion, sleep deprivation or limited recovery). In light of the unexpected sCorti patterns (also see Strahler and Ziegert, 2015), future studies should incorporate cross-sectional comparisons between police recruits, experienced officers and/or special forces and longitudinal designs to investigate the long-term effects of regular stress exposure in police officers: Do officers adaptively habituate to these critical incidents protecting performance delivery? Or do they show maladaptive, inadequate stress responses (e.g., hypocortisolism) resulting in performance decrements?

## Conclusion

In conclusion, police recruits exposed to reality-based shooting scenarios demonstrated greater increases in anxiety and mental effort in response to the HS scenario than the LS scenario. However, both scenarios elicited similar physiological responses (i.e., sAA secretion and decreases in cardiac vagal activity). Accordingly, shooting accuracy in both scenarios was relatively low and did not differ between the LS and HS scenario. Post-task cardiac vagal activity was directly linked to shooting performance. Specifically, lower post-task cardiac vagal activity was associated with better shooting performance. This strengthens the need to assess rest, reactivity, and recovery (the three Rs) of cardiac vagal activity (Laborde et al., 2017). In addition, high dispositional self-control was related to lower levels of anxiety, but impaired shooting performance in the LS scenario. Possibly, the attempt to control unwanted emotions and thoughts impairs the goal-directed focus on the task, resulting in performance decrements. Therefore, it is vitally important that police officers get to know and learn to accept psychological and physiological stress responses during reality-based training to ensure optimal task performance in HS situations. Using reality-based scenarios that are reflective of challenges individuals need to face in real life provides a comprehensive picture of psychological, physiological, and behavioral stress responses in critical incidents. This knowledge allows to refine effective police training programs, which in turn increases the security of police officers, suspects and civilians.

## Data Availability

The datasets generated for this study are available on request to the corresponding author.

## Ethics Statement

This study was carried out in accordance with the recommendations of the Declaration of Helsinki and under the supervision of experienced police instructors with written informed consent from all subjects. All subjects gave written informed consent in accordance with the Declaration of Helsinki. The protocol was approved by the Faculty of Behavioral and Cultural Studies, Heidelberg University, Germany.

## Author Contributions

In this interdisciplinary project, each author contributed to the study planning, data analysis, and interpretation with an additional focus on their respective area of competence. CK and MB were responsible for the biochemical analyses of the saliva samples. MF, CZ, AN, FE, and HP contributed crucially in drafting the aim of the study, concretizing the design, and finishing the manuscript. JR supervised the statistical analyses and the final draft of the manuscript. LG wrote the first draft of the manuscript and was essentially responsible for the statistical analyses and interpretation of the data, as well as for the communication between all authors during the development of the article.

## Conflict of Interest Statement

The authors declare that the research was conducted in the absence of any commercial or financial relationships that could be construed as a potential conflict of interest.
